# Sensitivity to Stroke Emerges in Kindergartners Reading Chinese Script

**DOI:** 10.3389/fpsyg.2017.00889

**Published:** 2017-06-02

**Authors:** Su Li, Li Yin

**Affiliations:** ^1^CAS Key Laboratory of Behavioral Science, Institute of Psychology, Chinese Academy of SciencesBeijing, China; ^2^Center for the Study of Language and Psychology, Department of Foreign Languages and Literatures, Tsinghua UniversityBeijing, China

**Keywords:** orthographic sensitivity, stroke, early reading, Chinese, kindergartner

## Abstract

To what extent are young children sensitive to individual stroke, the smallest unit of writing in Chinese that carries no phonological or semantic information? The present study examined Chinese kindergartners’ sensitivity to stroke and the contribution of reading ability and age to stroke sensitivity. Fifty five children from Beijing, including 28 4-year-olds (*M*_age_ = 4.55 years, *SD* = 0.28, 16 males) and 29 5-year-olds (*M*_age_ = 5.58 years, *SD* = 0.30, 14 males), were administered an orthographic matching task and assessed on non-verbal IQ and Chinese word reading. In the orthographic matching task, children were asked to decide whether two items were exactly the same or different in three conditions, with stimuli being correctly written characters (e.g., “
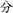
”), stroke-missing or redundant characters (e.g., “
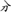
”), and Tibetan alphabets (e.g., “
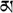
”), respectively. The stimuli were presented with E-prime 2.0 software and were displayed on a Surface Pro. Children responded by touching the screen and reaction time was used as a measure of processing efficiency. The 5-year-olds but not the 4-year-olds processed correctly written characters more efficiently than stroke-missing/redundant characters, suggesting emergence of stroke sensitivity from age 5. The 4- and 5-year-olds both processed correctly written characters more efficiently than Tibetan alphabets, ruling out the possibility that the 5 year olds’ sensitivity to stroke was due to the unusual look of the stimuli. Hierarchical regression analyses showed that Chinese word reading explained 10% additional variance in stroke sensitivity after having statistically controlled for age. Age did not account for additional variance in stroke sensitivity after having considered Chinese word reading. Taken together, findings of this study revealed that despite the visually highly complex nature of Chinese and the fact that individual stroke carries no phonological or semantic information, children develop sensitivity to stroke from age 5 and such sensitivity is significantly associated with reading experience.

## Introduction

The acquisition of reading skill is a crucial task in child development. Detection of the presence or absence of a stroke in a given character, e.g., “
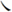
” in “
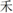
”, entails a combination of visual skills and orthographic knowledge, both of which play important roles in early reading development (e.g., visual skills: Huang and Hanley, 1995; Ho and Bryant, 1997; Siok and Fletcher, 2001; Valdois et al., 2004; McBride-Chang et al., 2011; Franceschini et al., 2012; Luo et al., 2013; Van der Leij et al., 2013; orthographic knowledge: Badian, 1994; Cassar and Treiman, 1997; Ho and Bryant, 1997; Lonigan et al., 2000; Cunningham et al., 2001; Li et al., 2006; Wang et al., 2015). The purpose of the current study is to examine to what extent young children are sensitive to individual stroke in a character, the smallest unit of writing in Chinese that carries no phonological or semantic information.

Children develop knowledge about the visual-orthographic characteristics of the writing they are exposed to before receiving formal literacy instruction (e.g., English: Lavine, 1977; Levy et al., 2006; Treiman et al., 2007; Pollo et al., 2009; Chinese: Ho et al., 2003; Luo et al., 2011; Yin and McBride, 2015). For example, 3-year-old English-speaking children accepted Latin letters as writing more often than visually dissimilar symbols such as Chinese characters (Lavine, 1977). Pre-phonological 4-year-olds from Brazil and United States produced spellings that reflect consistent differences in spelling pattern between Portuguese and English, indicating their implicit abstraction of regularities in the prints they are exposed to (Pollo et al., 2009). Chinese 5-year-olds learned to read significantly better when the subcomponents of character stimuli were legally positioned than when they were illegally positioned, when no phonetic cue was available, suggesting their implicit knowledge about the positional regularities in Chinese (Yin and McBride, 2015).

Most of the previous studies, however, examined children’s visual-orthographic knowledge at the level of word component, such as letter/letter string in alphabetic languages or character/radical (stroke combination that recurs across characters) in Chinese. Word components of such carry, in varying degrees, information of sound and meaning (although orthography, phonology, and semantics are intrinsically inseparable across writing systems). Few studies have examined whether children are sensitive to the elements within these components, e.g., the stroke “
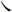
” in character “
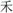
”, which carries no phonological or semantic information. The only study that examined young children’s awareness of stroke (Luo et al., 2011) asked children to discriminate between Chinese stroke and English letter in isolation, e.g., “
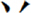
” and “f”. Stronger evidence for stroke sensitivity, however, should come from testing whether children can detect the removal of a single stroke from (e.g., “
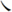
” in “
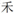
”) or the addition of a single stroke to (e.g., “
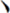
”in “
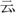
”) a real character.

In the present study, we investigated whether Chinese 4- and 5-year-old kindergarteners develop sensitivity to stroke in Chinese character and how such sensitivity is linked to reading ability and maturation.

Chinese orthography is known for high visual complexity. Words in alphabetic scripts are formed from a limited visual set (e.g., 26 letters in English and 22 in Hebrew), but the 1000s of characters in Chinese represent thousands of visually different stroke configurations. A stroke is a dot or a line written in one continuous movement (Anderson et al., 2013). As in character “
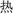
”, a dot can be in various directions (“
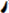
” or “
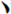
”) and a line can be horizontal (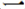), vertical (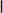), slanting (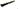), curved (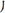) or contain a hook at the end (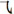). Around 80% of modern Chinese characters are compound characters composed of radicals, i.e., stroke patterns that recur across characters. The semantic radical (e.g., “
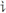
” in “
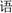
” and “
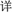
”) gives a clue to the character’s meaning and the phonetic radical (e.g., “
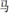
” in “
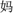
” and “
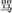
”) gives a clue to its pronunciation, though the cuing effect is not consistently reliable. Some radicals appear consistently in a fixed position in the character (e.g., “
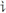
” always on the left, “
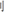
”always on the right, “
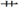
” always on the top). About 20% characters are simple characters that are not divisible into sub-components and thus are composed of individual strokes only (e.g., “
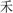
”).

Stroke sensitivity is important in learning to read Chinese. First, sensitivity to the identity and relative position of a stroke helps discriminate between characters that are visually similar but totally different in sound and meaning, which are abundant in Chinese (e.g., 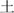 [/tu/, soil] and 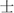 [/shi/, soldier], 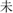 [/wei/, not] and 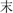
[/mo/, end], 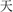 [/tian/, sky] and 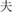 [/fu/, husband]). Second, it helps discover the internal structure of compound characters, identify radicals, and distinguish between visually similar radicals (e.g., “
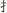
” and “
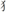
”, “
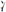
” and “
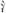
”); such abilities are important for recognition of Chinese characters (Shu et al., 2003).

We hypothesized that despite the high visual complexity of Chinese and the absence of linguistic information in stroke, Chinese kindergarteners may develop sensitivity to stroke before formally learning to read, and such sensitivity is significantly linked to reading ability in addition to maturational age. The hypotheses were based on a number of reasons.

First, orthographic processing is at least partly constrained by visual object processing that involves extraction of perceptual features of individual elements to identify visual objects (Grainger et al., 2012). With repeated exposure to letter combinations of manipulated frequency, baboons (non-human primates) learned to discriminate English words from non-words through extracting features of individual letters and their combinations. Characters represent a special class of visual objects exposed to Chinese children. There is evidence that Chinese children begin to pay more attention to the visual form information of words in highly familiar environment from age 5 (Zhao et al., 2014b).

Second, Chinese children seem to develop stronger visual skills than children learning to read alphabetic orthographies due to the visually demanding nature of Chinese orthography (Huang and Hanley, 1994; Demetriou et al., 2005; McBride-Chang et al., 2011). Chinese children showed clear advantage over British children on visual form discrimination skills (Huang and Hanley, 1994), and Chinese kindergartners outperformed Israeli and Spanish peers on task of visual spatial relationships (McBride-Chang et al., 2011). We reason that Chinese children’s better visual skills may facilitate stroke processing and may in fact reflect the consequence of stroke processing (Zhou et al., 2014).

Third, Chinese kindergartners have developed quite some knowledge about the visual-orthographic features of Chinese before receiving formal literacy instruction (Ho et al., 2003; Luo et al., 2011; Wang et al., 2015; Yin and McBride, 2015). In a character learning task (Yin and McBride, 2015), the 4-year-olds learned pseudocharacters (character stimuli consisting of real radicals placed in legal positions) and non-characters (character stimuli consisting of real radicals placed in illegal positions) significantly better than random stroke combinations, suggesting their structural knowledge of characters; the 5-year-olds learned pseudocharacters significantly better than non-characters, reflecting their knowledge of the identities and positional regularities of radicals. Luo et al. (2011) found that Chinese 5-year-olds could discriminate between Chinese stroke and English letter when presented in isolation (e.g., 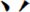 and f). In the present study, we tested Chinese 4- and 5-year-olds’ stroke sensitivity at a much finer level, examining whether children can detect the removal or addition of one stroke from a real character (e.g., “
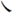
” in “
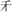
”, or “
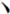
” in “
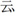
”).

Finally, reading ability modulates visual expertise for word processing across orthographies (Burgund et al., 2006; Li et al., 2013; Zhao et al., 2014a; Su et al., 2015). Burgund et al. (2006) found the emergence of letter specific-processing in English is linked to increased reading skill rather than increased age among a group of 6-to-19-year-olds. Zhao et al. (2014a) found that fine neural tuning for visual words, which reflects sensitivity to orthographic regularity, emerged in 7-year-old German-speaking children with high but not low reading ability. Li et al. (2013) found that neural specialization for word processing is significantly influenced by reading experience (indexed by sight vocabulary) in 5-and 6-year-old Chinese kindergartners.

In the present study, we tested a group of 4 and 5-year-old kindergarten children in Beijing who have not received formal literacy instruction. We used an orthographic matching task to tap children’s sensitivity to stroke in character. There were three orthographic conditions, using stimuli of correctly written characters, stroke missing or redundant characters, and Tibetan alphabets, respectively. In each condition there were 20 stimulus pairs, half of which were the same and half of which were different. Using a Surface Pro with E-Prime 2.0 software to present stimuli pairs, we asked children to decide whether the two items on the screen were exactly the same or different by touching the corresponding happy or sad face. This design allowed us to analyze children’s reaction time as a measure of processing efficiency, which was more objective and reliable than oral reports, as were typically used in previous research with young children.

If children were sensitive to stroke, they would process correctly written characters more efficiently than stroke missing/redundant characters. To rule out the possibility that children’s lower efficiency in processing stroke missing/redundant characters is due to foreignness of the stimuli’s look (e.g., 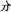) rather than sensitivity to the missing/redundant stroke, we added the condition of Tibetan alphabets because Tibetan alphabet looks very different from Chinese character both in terms of the shape of its constituents and the configuration matter of its constituents (e.g., 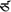). Children who were sensitive to stroke should show lower efficiency in processing both Tibetan alphabets and stroke-missing/redundant characters compared with correctly written characters, whereas children who have not yet developed sensitivity to stroke may show lower efficiency in processing Tibetan alphabets but not stroke-missing/redundant characters compared with correctly written characters.

We also assessed children’s Chinese word reading ability. Based on findings from previous studies, we predicted a unique contribution of reading ability to the emergence of stroke sensitivity. In view of the young age of the participants in the current study (4-and 5-year-olds) and the more demanding nature of the task (processing the smallest unit in almost the most visually complex orthography- Chinese), we might also expect that maturation play an equally important role in the emergence of stroke sensitivity.

## Materials and Methods

### Children

Fifty five children from Beijing participated in the study. They were 28 4-year-olds (*M*_age_ = 4.55 years, *SD* = 0.28, 16 males) and 29 5-year-olds (*M*_age_ = 5.58 years, *SD* = 0.30, 14 males). All children were native Chinese speakers and had normal vision and no known disorders. The researchers explained the purpose and procedure of the study to children’s parents/guardians and obtained consents from all participating children’s parents/guardians. Children were allowed to withdraw at any time of the study and their rights and privacy were protected throughout the study according to the American Psychological Association Ethical Principles of Psychologists and Code of Conduct, Including 2010 Amendments^1^.

### Materials

The experimental materials included three types of stimuli: correctly written characters, stroke-missing/redundant characters, and Tibetan alphabets. There were 20 items for each type of stimulus. As shown in **Table 1**, the correctly written characters were common characters which included five simple characters, eight left–right structured compound characters, and seven bottom–up structured compound characters. The number of the three structures was based on their distributions in the total number of Chinese characters (Dictionary of Chinese Character Information, 1988, as cited in Shu et al., 2003). The average frequency of the correctly written characters was 55985.5 per 10 millions (range: 22795.5–379917), and the average number of the strokes was 6 (range: 2–10). The stroke-missing/redundant characters were constructed by adding or deleting one stroke from the correctly written characters, with half of them made from adding one stroke to the correctly written characters and the other half made from deleting one stroke from the correctly written characters. The Tibetan alphabet letters were selected randomly from the 36 consonant letters in Tibetan. The average number of the letter strokes was 4 (range: 3–6). While we were primarily concerned with the performance difference between correctly written characters and stroke-missing/redundant characters, we compared performance on correctly written characters and Tibetan alphabet letters as well in order to elucidate the difference between correctly written characters and stroke-missing/redundant characters. All stimuli were presented in 180-point regular script font with the same size of 300 × 350 pixels. All stimuli were presented centrally on the screen, in black against a white background.

**Table 1:** Samples of the three types of stimuli used in the orthographic matching task.

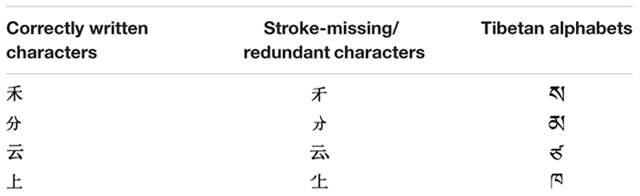

### Tasks

#### General Cognitive Ability Measurement

We administered the Combined Raven’s Test (CRT-Chinese version, 1991) to assess children’s IQ. The internal consistency reliability for this task was 0.86.

#### Orthographic Matching Task

**FIGURE 1:**
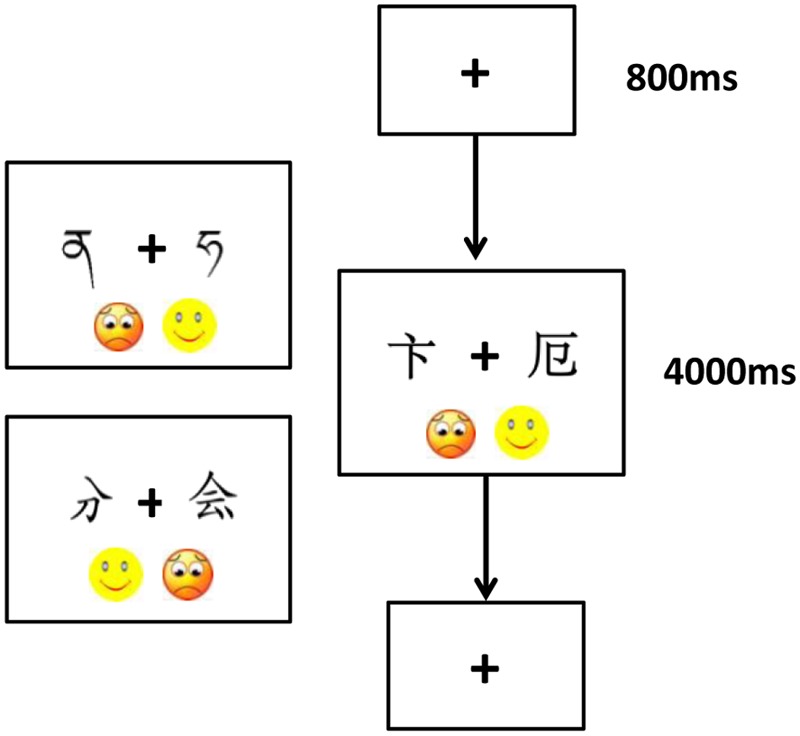
Exemplar of one trial in the orthographic matching task (the two pictures on the left indicate the two categories of stimuli).

We used an orthographic matching task to tap children’s sensitivity to strokes. There were three orthographic conditions using stimuli of correctly written characters, stroke-missing/redundant characters, and Tibetan alphabets, respectively. As shown in **Figure 1**, in each condition, there were 20 stimulus pairs, half of which were exactly the same and half of which were different. We used E-prime 2.0 software to present the stimuli and used a Surface Pro to display the stimulus pairs. Children responded by touching the screen so that we could obtain their direct response to the stimulus pair. The pairing of stimuli, the left–right positioning of stimulus in the pair, and the order of pair presentation were randomized in each condition. The order of condition was counterbalanced across children.

In each trial (**Figure 1**), following a 800 ms fixation, each stimulus pair was presented until the children made response. Children were instructed to decide whether the two items on the screen were exactly the same or different and to indicate their response as quickly as possible by using their index finger of the right hand to touch the happy face (“
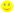
”) or sad face (“
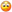
”) on the screen. Touching the happy face indicated a “same” response and touching the sad face indicated a “different” response. The left–right positioning of the two faces was counterbalanced across children. Six practice trials were provided before the formal experiments began to ensure that children understood how to perform in the task.

We used a “catching butterfly” play to obtain the basic reaction time of each child. Children were asked to touch as quickly as they could a black butterfly that appeared in the middle of the screen. The play was presented through E-prime 2.0 software on the Surface Pro as well. The presenting time of each butterfly was 2000 ms and the reaction time widows were between 2000 and 8000 ms with 1500 ms as a jitter. The picture of butterfly would disappear after children touched it. The play contained 12 trials including two practice trials.

#### Chinese Word Reading Task

Children were asked to read aloud 50 Chinese single-character words presented in order of increasing difficulty (Wang et al., 2015). Testing was discontinued when children failed to read 10 characters consecutively. One point was given for each correctly read item. The maximum score was 50. The internal consistency reliability for this task was 0.96.

### Procedure

Children completed four tasks over a period of 2 weeks in the first semester of the school year. All tasks were administered individually by trained graduate students in a quiet reading room. Half of the children in each age group completed the orthographic matching task, preceded by the basic reaction time measurement, in the first week, and the Raven’s test and the Chinese word reading task in the second week; the other half of children completed the Raven’s test and the Chinese word reading task in the first week, and the orthographic task preceded by the basic reaction measurement in the second week. The orthographic matching task lasted 20–25 min, with a 5-min break in the middle. The Raven’s test and the Chinese word reading task took approximately 10 min each.

## Results

### Descriptive Statistics

**Table 2** shows children’s performance (raw score) in each task as a function of age group. In the orthographic matching task, the accuracy rate of the 4-year-olds was 0.96, 1.00, and 0.94 for the correctly written characters, stroke-missing/redundant characters, and Tibetan alphabets, respectively, with no significant difference across conditions, *F*(2,81) = 2.00, *p* = 0.14; similarly, the accuracy rate of the 5-year-olds was 1.00, 0.97, and 0.96 for the correctly written characters, stroke-missing/redundant characters, and Tibetan alphabets, respectively, with no significant difference across conditions, *F*(2,84) = 1.22, *p* = 0.30. Given that the task was designed to be easy for young children to understand and perform (children were just to decide whether the two stimuli on the screen were same or not at the perceptual level), the high accuracy rate across conditions was expected, indicating that the participants performed well and the data was reliable. We then focused on reaction time of the correct responses as a measure of processing efficiency, i.e., an index of sensitivity, in subsequent analyses. Reactions times that were over two standard deviations away from the mean in a given condition in each group were removed from the data. Overall, 1.8% of the total 4860 trials were removed from the data for this reason.

**Table 2:** Mean (standard deviation) of age, basic reaction time, reaction time in orthographic matching, IQ, and Chinese word reading as a function of age group.

	Age group		Group difference *F*(1,55)
	4 year olds	5 year olds	
*N*	28	29	
Age (in year)	4.55 (0.27)	5.58 (0.29)	187.52^∗∗∗^
Basic reaction time	944.57 (263.80)	741.62 (154.49)	12.58^∗∗^
Reaction time in orthographic matching			
*Correctly written character*	2653.10 (519.25)	2130.26 (282.69)	22.50^∗∗∗^
*Stroke-missing/redundant character*	2671.13 (656.37)	2333.87 (396.99)	5.55^∗^
*Tibetan alphabet*	2755.47 (649.14)	2332.41 (396.68)	8.89^∗∗^
IQ	10.89 (3.53)	15.00 (3.86)	17.06^∗∗∗^
Chinese word reading	8.46 (12.32)	16.17 (14.08)	4.71^∗^

*^∗∗∗^*p* < 0.001, ^∗∗^*p* < 0.01, ^∗^*p* < 0.05.*

**FIGURE 2:**
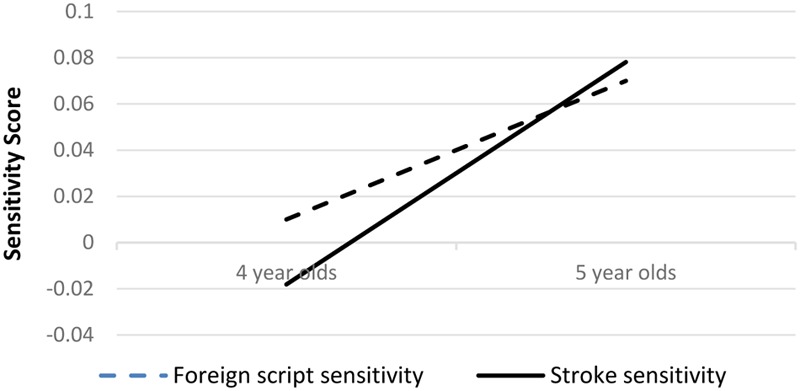
Sensitivity scores for the 4-year-olds and the 5-year-olds, respectively. Stroke sensitivity score was computed by subtracting the correctly written character score from the stroke-missing/redundant character score and dividing by the stroke-missing/redundant character score. Foreign script sensitivity score was computed by subtracting the Tibetan alphabet score from the correctly written character score and dividing by the Tibetan alphabet score.

Following Burgund et al. (2006), to reduce spurious distortions of within-subject differences due to overall group differences (Chapman and Chapman, 1973; Chapman et al., 1994), we computed two sensitivity scores in the following manner for each child. Stroke sensitivity score was computed by subtracting the correctly written character score from the stroke-missing/redundant character score and dividing by the stroke-missing/redundant character score ([stroke-missing/redundant character score-correctly written character score]/stroke-missing/redundant character score). Foreign script sensitivity score was computed, for purpose of discriminating stroke sensitivity from sensitivity caused by the foreign or unusual look of stimuli, by subtracting the Tibetan alphabet score from the correctly written character score and dividing by the Tibetan alphabet score ([Tibetan alphabet score- correctly written character score]/Tibetan alphabet score). **Figure 2** shows the scores of stroke sensitivity and foreign script sensitivity in each group.

Before analyzing, we checked the normality of distribution of the data. Shapiro–Wilk tests showed that the data of foreign script sensitivity was normally distributed in both age groups, *p*s > 0.05, and the data of stroke sensitivity was normally distributed in the 5-year-olds, *p* = 0.64, but not in the 4-year olds, *p* = 0.002. Considering the relatively small sample size in the current study and that parametric analysis of transformed data is a better strategy than non-parametric analysis because it appears to be more powerful than the latter (Rasmussen and Dunlap, 1991), we normalized the data of stroke sensitivity and foreign sensitivity using rank-case transformation, which was reported as working better for small sample size in association tests than logarithm and Box-Cox transformations (Goh and Yap, 2009). We used the normal scores obtained using Blom’s formula as dependent variables in the following analyses.

### Development of Stroke Sensitivity

Taking basic reaction time as the covariate and age group as the fixed effect, univariate analysis was conducted for stroke sensitivity and foreign script sensitivity, respectively. Homogeneity of the covariate coefficient was checked first. For both stroke sensitivity and foreign script sensitivity, the interaction between age group and basic reaction time was not significant, *p*s > 0.12, indicating that homogeneity of the covariate coefficient across age groups could be assumed in both analyses.

For stroke sensitivity, the 5-year-olds were significantly higher than the 4-year-olds, *F*(1,54) = 6.89, *p* = 0.011, $\eta_{p}^{2}$ = 0.11. The 5-year-olds processed correctly written characters (e.g., “
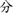
”) more efficiently, as reflected by the positive score of stroke sensitivity, than stroke-missing/redundant characters (e.g., “
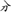
”), whereas the 4-year-olds did not. For foreign script sensitivity, no significant group difference was found, *F*(1,54) = 0.15, *p* = 0.70. The 5 year olds and the 4 year olds both processed more efficiently, as reflected by the positive scores, correctly written characters than Tibetan alphabets (e.g., “
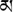
”).

### Contribution of Age and Reading Ability to Stroke Sensitivity

Across age groups and with basic reaction time statistically controlled, partial correlation was conducted among stroke sensitivity, age (expressed in months as a continuous variable), non-verbal IQ, and Chinese word reading. Stroke sensitivity was significantly correlated with age, *r* = 0.37, *p* = 0.005, and Chinese word reading, *r* = 0.42, *p* = 0.001, but not with non-verbal IQ, *p* = 0.169.

To better understand the relative contribution of age (maturation) and word reading ability (reading experience) to stroke sensitivity, hierarchical regression analyses were conducted with the dependent variable being stroke sensitivity and the independent predictors being age and Chinese word reading entered in different steps. We first examined whether the assumptions of regression were met, namely, linearity, normality, homoscedasticity, and independence. Visual inspection of the P-P plot confirmed the normality of error distribution. Examination of the scatter plot of residuals confirmed the linearity of relationship and inspection of the plot of residuals versus predicted values confirmed the constancy of variance of the errors (homoscedasticity). The Durbin-Watson statistic being 2.37 (with 1.4–2.6 considered being ideal) supported the statistical independence of the errors. We then ran the hierarchical regression models. In model 1, Chinese word reading was entered in the first step and age was entered in the second step. In model 2, age was entered in the first step and Chinese word reading was entered in the second step. **Table 3** shows the results of the final models. In model 1, after statistically controlling for Chinese word reading, age did not explain significant additional variance in stroke sensitivity, *r*^2^ change = 0.04, *F*-change (1,54) = 2.63, *p* = 0.11. In model 2, after having statistically controlled for age, Chinese word reading explained significant additional 10% of variance in stroke sensitivity, *F*-change (1,54) = 6.89, *p* = 0.01.

**Table 3:** Results of the final models predicting stroke sensitivity from age and reading ability.

Model	Predictors	*R^2^*	*R^2^* change	*B*	*SE B*	β
1	Step 1: Chinese word reading	0.18	0.18^∗∗^	0.02	0.01	0.34^∗^
	Step 2: Age	0.22	0.04	0.35	0.22	0.21
2	Step 1: Age	0.12	0.12^∗^	0.35	0.22	0.21
	Step 2: Chinese word reading	0.22	0.10^∗^	0.02	0.01	0.34^∗^

*^∗∗^p < 0.01, ^∗^*p* < 0.05.*

## Discussion

The present study examined 57 4-and 5-year-old Chinese children’s sensitivity to stroke, the smallest unit of writing that carries no phonological or semantic information in Chinese. The stroke-level sensitivity were assessed through an orthographic matching task in which children were asked to judge whether two items displayed on a Surface Pro were exactly the same or different. Importantly, we recorded and analyzed children’s reaction time, which was more objective and reliable than oral reports. Also, we explored the association of age and reading experience to the emergence of stroke sensitivity.

We found that stroke-level sensitivity emerges from age 5. The 5-year-olds processed stroke-missing/redundant characters more slowly than that of correctly written characters. Also, their stroke sensitivity score was significantly higher than that of the 4-year-olds. Importantly, the 5-year-olds did not demonstrate sensitivity to foreign script (Tibetan alphabets), indicating that their sensitivity to stroke was not due to the unusual look of the stroke-missing/redundant characters. Children with stroke sensitivity demonstrated poorer performance, in this study, on the stroke-missing/redundant character task than those without stroke sensitivity, which was intended and reflected in terms of slower processing (as indexed by longer reaction time) rather than lower accuracy rate. In other words, the “poorer” performance of children with stroke sensitivity on the stroke-missing/redundant characters does not mean that they cannot distinguish correctly written characters and stroke-missing/redundant character; rather, it means that children who are sensitive to strokes within the character are more easily disturbed (than children who have not yet developed such sensitivity) when they process stroke-missing/redundant character and thus take longer reaction time. This is in line with previous findings from studies examining letter processing in children from alphabetic writing systems. Developmental work on letter processing has demonstrated that while children become faster at processing both letters and non-letters with age (Gibson et al., 1962; Reitsma, 1978), children demonstrate improved performance for letters compared to non-letters as young as 6 years of age (Miller and Wood, 1995; Burgund et al., 2006). These results are also consistent with findings from Zhao and Li (2014) in which the researchers used a lexical decision task with four types of stimuli (Chinese characters, stroke combinations, character-like line drawings with stroke features removed, and general line drawings) and asked Chinese 3- to-6-year-olds to judge whether the stimulus was a real character or not. They found that children’s awareness of stoke developed very fast during 4–5 years and reached peak at age 5. The present study supported findings from Zhao and Li (2014) but provided more objective, thus stronger, evidence for the emergence of stroke-level sensitivity from age 5 in Chinese children.

We also found that stroke sensitivity was linked more to reading experience than to maturation. Results of hierarchical regression analyses showed that Chinese word reading explained significant additional variance in stroke sensitivity after having statistically controlled for age, but age did not explain significant additional variance in stroke sensitivity beyond reading experience. This finding is consistent with previous studies showing that reading experience, rather than age, plays a more important role in learning to read among school-age children (e.g., Burgund et al., 2006; Zhao et al., 2014a). We expected that at an earlier stage of learning to read, e.g., in kindergarten years, maturation should play an equally important role, considering that perception of objects and events in the natural environment is a real-time task and a child needs to have mature sensory primitives (Aslin and Smith, 1988), but finding of the current study did not support this expectation. We found that although the young children’s retinal processes is still developing, their visual acuity enables them to process fine features of written words (Gibson et al., 1962), e.g., stroke, the finest orthographic unit in the visually highly complex Chinese characters, and such sensitivity is significantly associated with their reading experience.

The current study sheds important light to the nature of early orthographic knowledge development. Similar to the alphabetic-language speaking counterparts(e.g., Lavine, 1977; Levy et al., 2006; Treiman et al., 2007; Pollo et al., 2009), Chinese kindergartners develop sensitivity to the identity and position of the components of writing they are exposed to before receiving formal literacy instruction. Different from previous studies that mostly examined components of writing that carry phonological or semantic information in varying degrees, the current study, for the first time to our knowledge, investigated beginning readers’ sensitivity to the smallest unit of writing that carries no linguistic information at all in almost the most visually complex orthographies in the world. Our finding that children as young as age 5 can detect removal or addition of a single stroke from a character provides enlightening evidence that children develop formal knowledge about writing (visual-graphic and orthographic) independent of functional knowledge (phonological or semantic), and that visual processing of the holistic features of writing (which enables detection of a single change of stroke in a whole character in the present study) is an important aspect of orthographic knowledge, especially in the early stage of learning to read.

Learning to read involves developing visual expertise for written form of words and linking this visual information to phonological and semantic information of words. It is the first step of visual-orthographic processing that is crucial for reading and learning to read (Maurer and McCandliss, 2007). Recent electrophysiological studies showed that visual expertise for written words correlates with children’s individual reading ability (Zhao et al., 2014a) and that visual word expertise (written word N1) can be observed in young children who has not received formal reading training (Li et al., 2013). It was also reported that dyslexic children showed reduced N1 tuning for written words (Maurer et al., 2007). In future research, it is intriguing to explore early behavioral predictors, such as sensitivity to stroke, for later visual word expertise development. Such studies will help eventually elucidate the developmental and neural mechanisms underlying reading development.

## Ethics Statement

This study was carried out in accordance with the recommendations of Tsinghua University Research Ethics Committee. All subjects gave written informed consent from their parents.

## Author Contributions

SL and LY collaboratively worked on the conception of the study, the acquisition, analysis, and interpretation of the data, and the writing-up of the paper.

## Conflict of Interest Statement

The authors declare that the research was conducted in the absence of any commercial or financial relationships that could be construed as a potential conflict of interest.

## Footnotes

1 http://www.apa.org/ethics/code/index.aspx
